# Supplementary material for: Maternal regulation of biliary disease in neonates via gut microbial metabolites
Source: Nat Commun. 2022 Jan 10;13:18. doi: 10.1038/s41467-021-27689-4 (PMC8748778; doi:10.1038/s41467-021-27689-4)
Supplement: Supplementary file 1 — Supplementary Information [file 41467_2021_27689_MOESM1_ESM.pdf]

Supplementary Information for

Maternal regulation of biliary disease in neonates via gut microbial metabolites

Jai Junbae Jee†, Li Yang†, Pranavkumar Shivakumar†, Pei-pei Xu†, Reena Mourya, Unmesha Thanekar, Pu Yu, Yu Zhu, Yongkang Pan, Haibin Wang, Xufei Duan, Yongqin Ye, Bin Wang, Zhu Jin, Yuanmei Liu, Zhiqing Cao, Miki Watanabe-Chailland, Lindsey E. Romick-Rosendale, Michael Wagner, Lin Fei, Zhenhua Luo, Nicholas J. Ollberding, Shao-tao Tang*, Jorge A Bezerra*

*Correspondence to: jorge.bezerra@cchmc.org (J.A.B.) and tshaotao83@hust.edu.cn (S.T.)

†Those authors have equal contributions to this project.

This PDF file includes:

Figs. S1 to S10

Supplementary Figure 1 (S1)

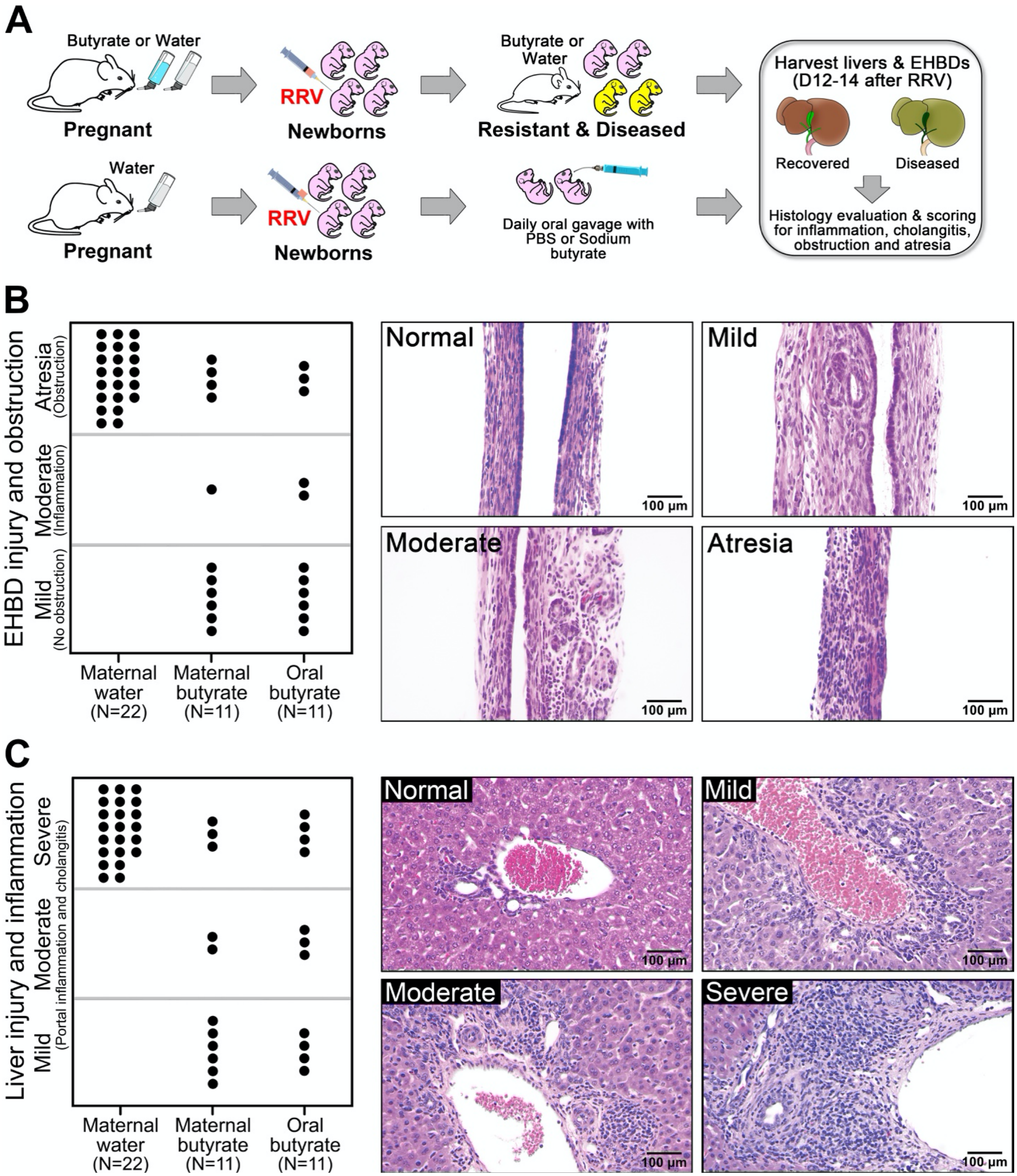

Fig. S1. Decrease in bile duct and liver injury following passive or active exposure to butyrate.

(**A**) Schematic diagram showing evaluation of liver and extrahepatic bile duct (EHBD) histology in RRV infected newborns born to butyrate or water-fed dams, with a subgroup of newborn mice also receiving sodium butyrate. (**B** and **C**) Histological assessment of EHBDs and livers using sections stained with hematoxylin–eosin. For EHBDs, sections were obtained along the entire length of the duct to enable the accurate assessment of the extent of periductal inflammation, intralumenal plugging, subepithelial inflammation, and complete or segmental atresia of the bile duct. Livers were scored based on the grade of portal inflammation and cholangitis. Histology panels on the right show gradation of EHBD tissue injury and liver inflammation. Filled circles depict individual mice. N=22 (water-fed mothers), N=11 (butyrate-fed mothers), N=11 (oral butyrate-fed neonates). 15-30 EHBD and 5-10 liver sections (corresponding to >100 sections at 200x or 400x magnification fields) stained with H&E per tissue specimen were evaluated for histology analysis. Source data for this figure are provided as a Source Data file.

Supplementary Figure 2 (S2)

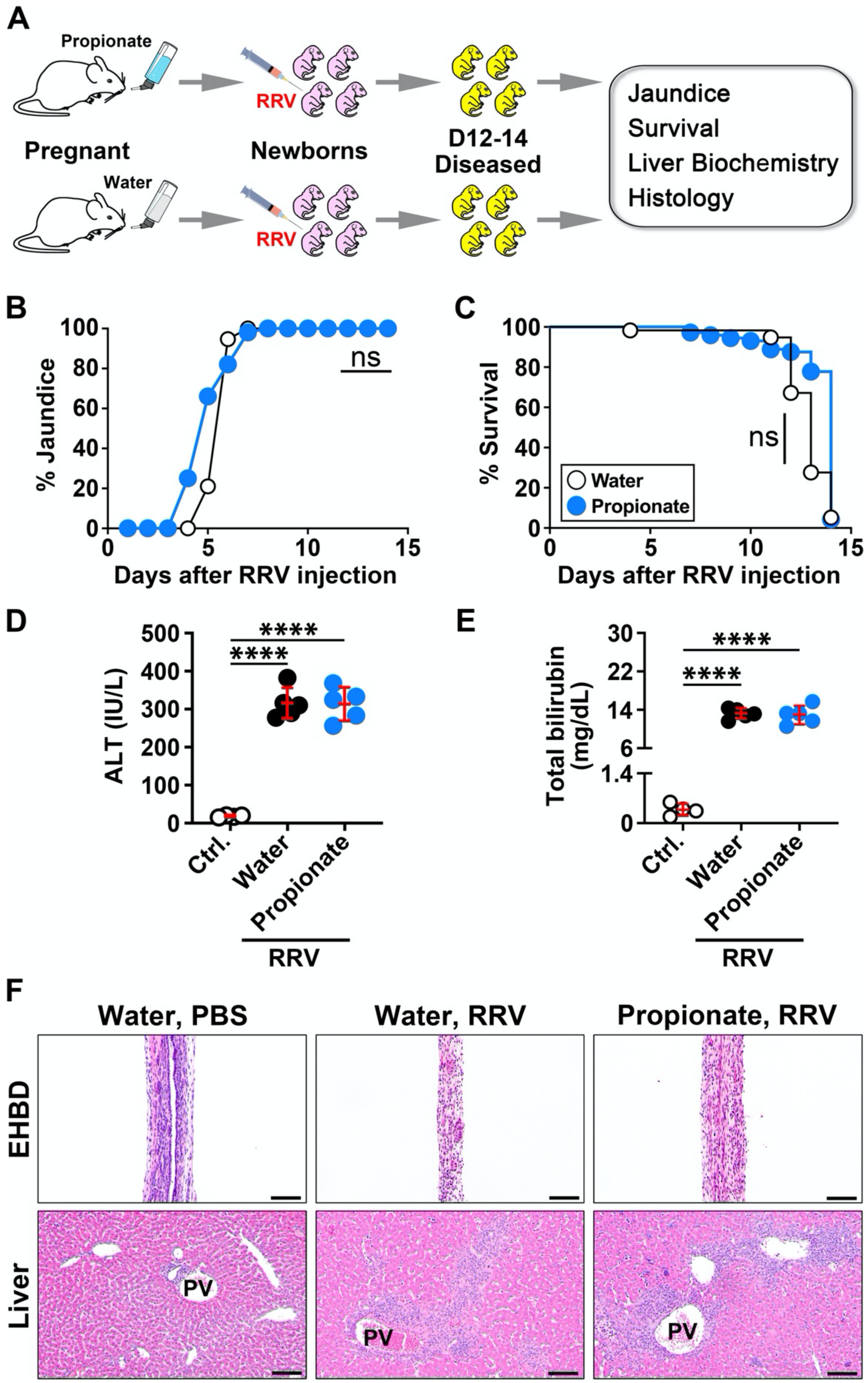

Fig. S2. Neonatal mice from propionate-fed mothers show persistent liver injury and bile duct obstruction.

(**A**) Schematic representation of propionate or water administration followed by evaluation of indicators of tissue injury, disease phenotype, and histomorphology of rotavirus (RRV)-infected mice. Panel (**B**) depicts incidence of jaundice (generalized linear mixed effect model with logit link and two-sided Wald test with Bonferroni correction; ns=not significant) and (**C**) shows survival (two-sided log-rank test; ns=not significant) rates of RRV-infected newborn mice from water- or propionate-fed mothers (n=15-22 mice per group). (**D**) Plasma alanine aminotransferase (ALT) and total bilirubin (**E**) from newborn mice 12-14 days after phosphate buffered saline (PBS, Ctrl; n=4) or RRV from water- or propionate-fed mothers (Mean ± SD, two-tailed ANOVA with Duncan's multiple comparison, n=5 per group from water- or propionate-fed mothers). (**F**) Extrahepatic bile duct (EHBD) and liver sections 12-14 days after PBS or RRV (water and propionate = maternal feeding; magnification bar=100µm; PV=portal vein). 15-25 EHBD and 5-10 liver sections (corresponding to >50 sections at 200x or 400x magnification fields from n=10-17 mice) stained with H&E per tissue specimen were evaluated for histology analysis. Source data for this figure are provided as a Source Data file.

Supplementary Figure 3 (S3)

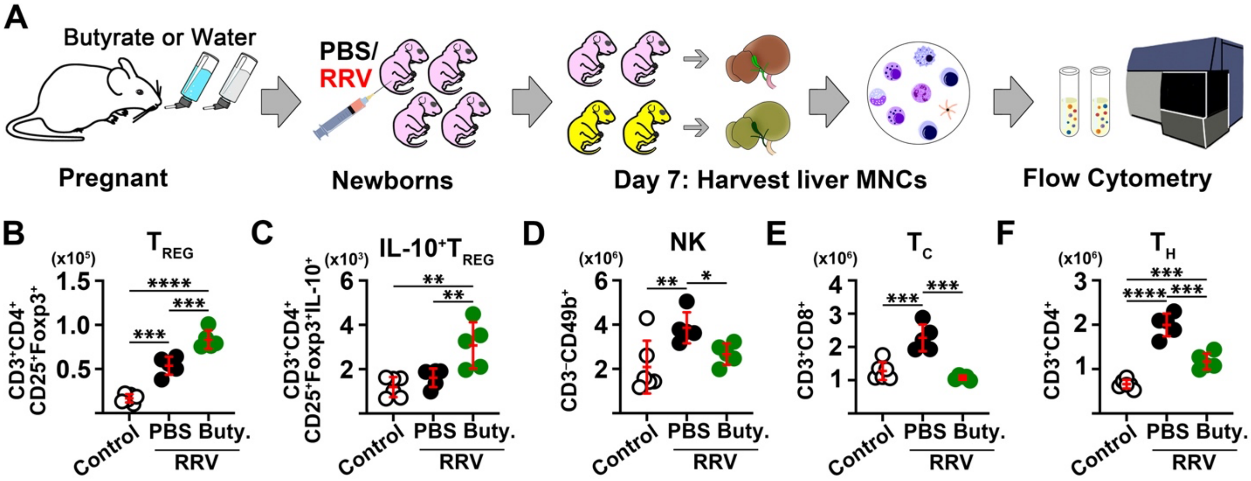

Fig. S3. Neonatal mice of butyrate-fed mothers show decreased effector immune cells and increased hepatic TREG populations.

(**A**) Diagrammatic representation of mononuclear cell harvest from livers and flow cytometry analysis. (**B**,**C**) Flow cytometry analysis of TREG and IL-10^{+}TREG immune cell populations in total hepatic mononuclear cells from day 7 saline-control and RRV-infected neonatal mice from water- or butyrate-fed mothers (Mean ± SD, two-tailed ANOVA with Duncan's multiple comparison; n=5-6 independent neonatal mice per group. ***p*<0.01, ****p*<0.001, *****p*<0.0001). (**D-F**) Hepatic NK, CD8^{+}, and CD4^{+} T cells in livers of newborn mice from control and water or butyrate-fed mothers identified by flow cytometric analyses (Mean ± SD, two-tailed ANOVA with Duncan's multiple comparison; n=5-6 independent neonatal mice per group. **p*<0.05, ***p*<0.01, ****p*<0.001, *****p*<0.0001). The gating strategy for flow cytometric analysis is shown in Fig. S10A-G. Source data for this figure are provided as a Source Data file.

Supplementary Figure 4 (S4)

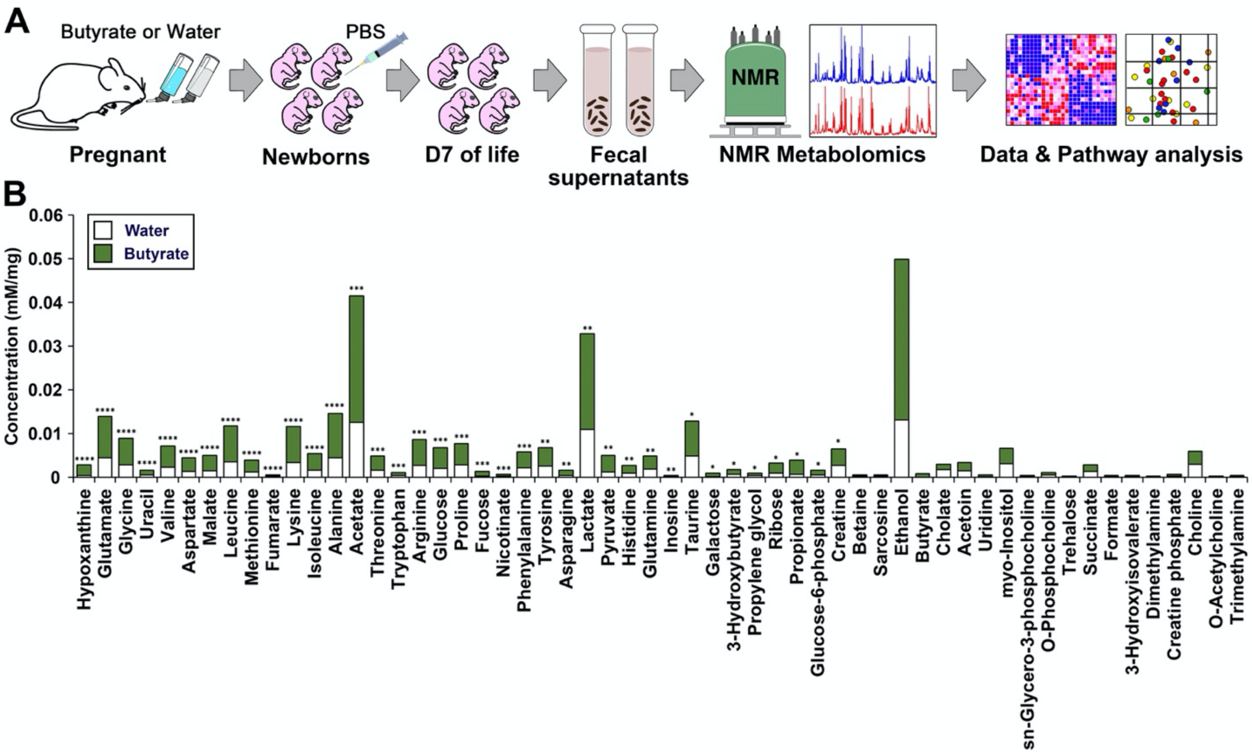

Fig. S4. Fecal metabolite composition in newborns from water- or butyrate-fed mothers. (**A**) Schematic view of NMR metabolomics and data analysis from neonatal fecal supernatants. (**B**) Proton nuclear magnetic resonance (^{1}H-NMR) metabolomics analysis of murine fecal supernatants quantified a total of 56 compounds. Stacked bar graph comparing means of normalized metabolite concentrations in fecal supernatants of neonatal mice from water- and butyrate-fed mothers, unpaired Student's t-test with two-tailed distribution, n=7-9 per group; **p*<0.05; ***p*<0.01; ****p*<0.001; *****p*<0.0001. Source data for this figure are provided as a Source Data file.

Supplementary Figure 5 (S5)

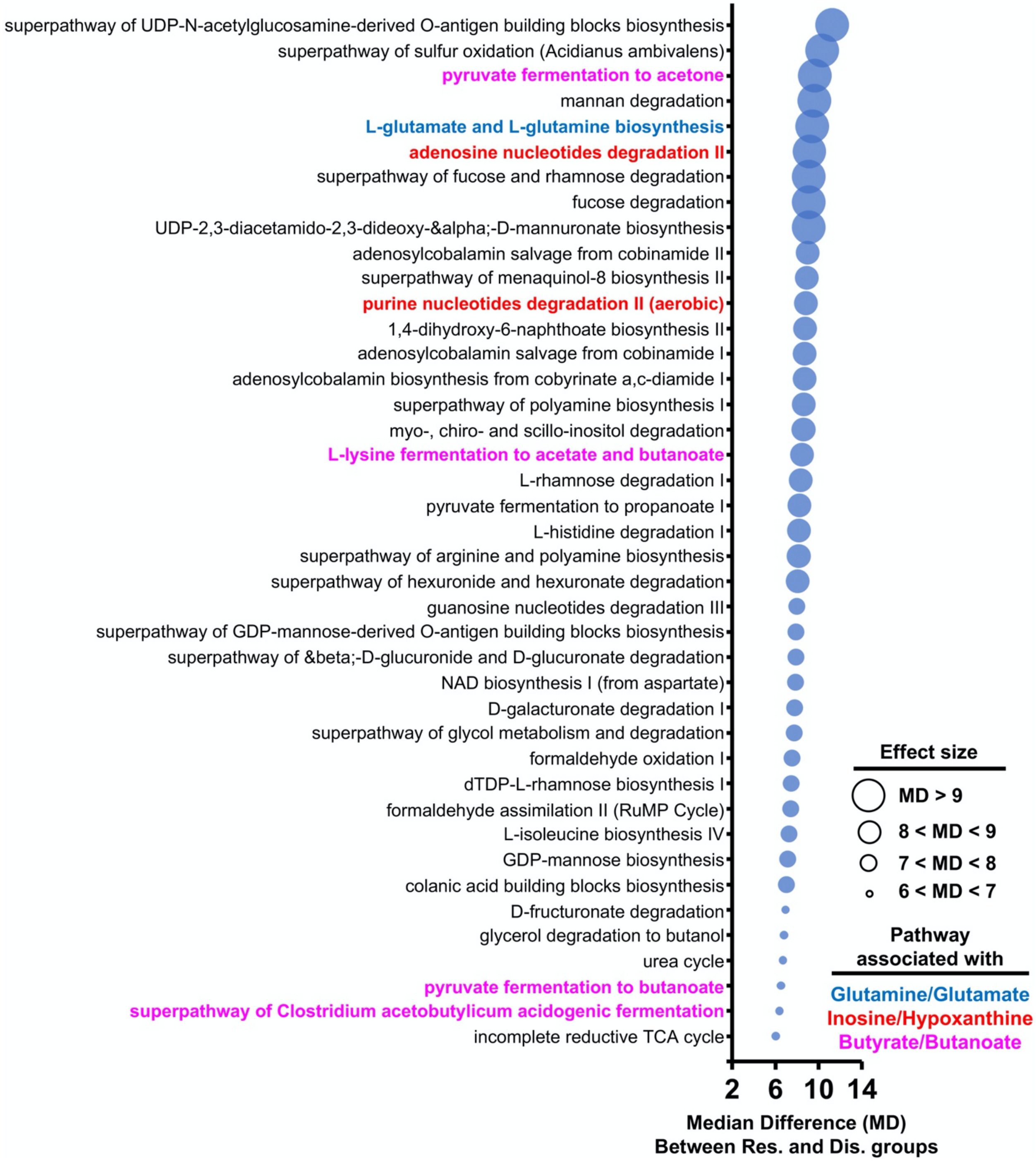

Fig. S5. Enrichment of pathways in fecal microbiome analyses from diseased and disease-resistant newborn mice.

Graphical representation of functional pathways significantly abundant in resistant compared to diseased neonates born to butyrate-treated dams. Functional pathways were inferred by MetaCyC pathways using Phylogenetic investigation of communities by reconstruction of unobserved states (PICRUSt2) v2.3.0 beta analytical tool and 16s rRNA sequencing data of resistant (N=6) and diseased neonates (N=11, adjusted p-value < 0.05). The p-values were obtained by two-sided Wilcoxon rank-sum test and adjusted for multiple testing corrections using the Benjamini-Hochberg method within the R package ANOVA-like differential gene expression version 2 (ALDEx2) version 1.22.0. Source data for this figure are provided as a Source Data file.

Supplementary Figure 6 (S6)

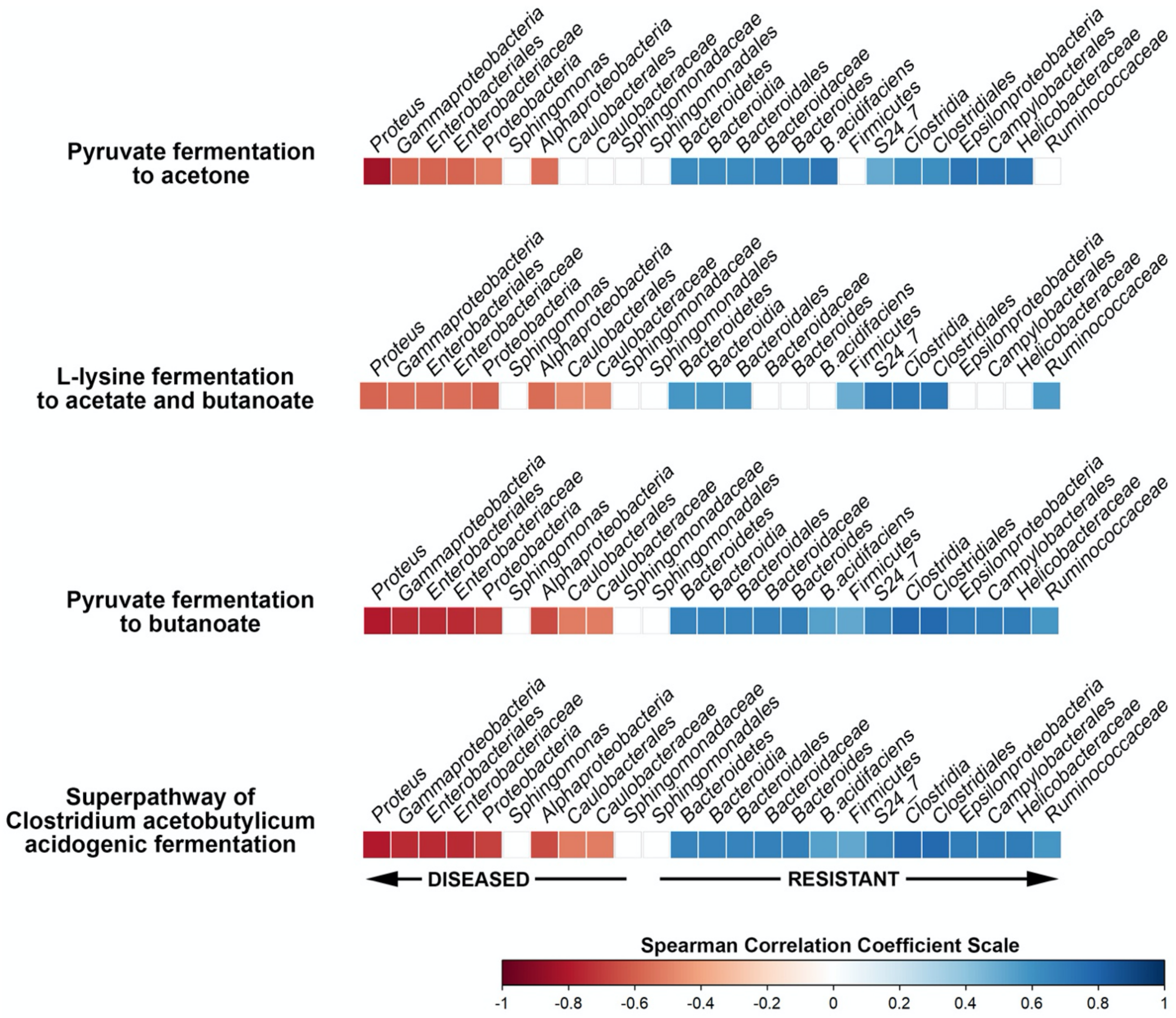

Fig. S6. Representation of butyrate pathways in bacterial taxa from diseased and resistant groups of mice.

Graphical representation of the strength of butyrate/butanoate pathways associated with bacterial taxa in RRV-infected diseased and resistant groups of neonates born to butyrate-treated dams. The four butyrate pathways were inferred by MetaCyC pathways (Fig. S5) and correlated with the bacterial taxa signatures depicted in the cladogram of Figure 3G. Spearman's correlation coefficients, p-values and correlogram were obtained for differentially abundant taxa and

MetaCyc pathways using the *rstatix* module in the R package: cor_mat(method="spearman"), cor_get_pval() and cor_plot(method = "color",insignificant = "blank"), respectively. Source data for this figure are provided as a Source Data file.

Supplementary Figure 7 (S7)

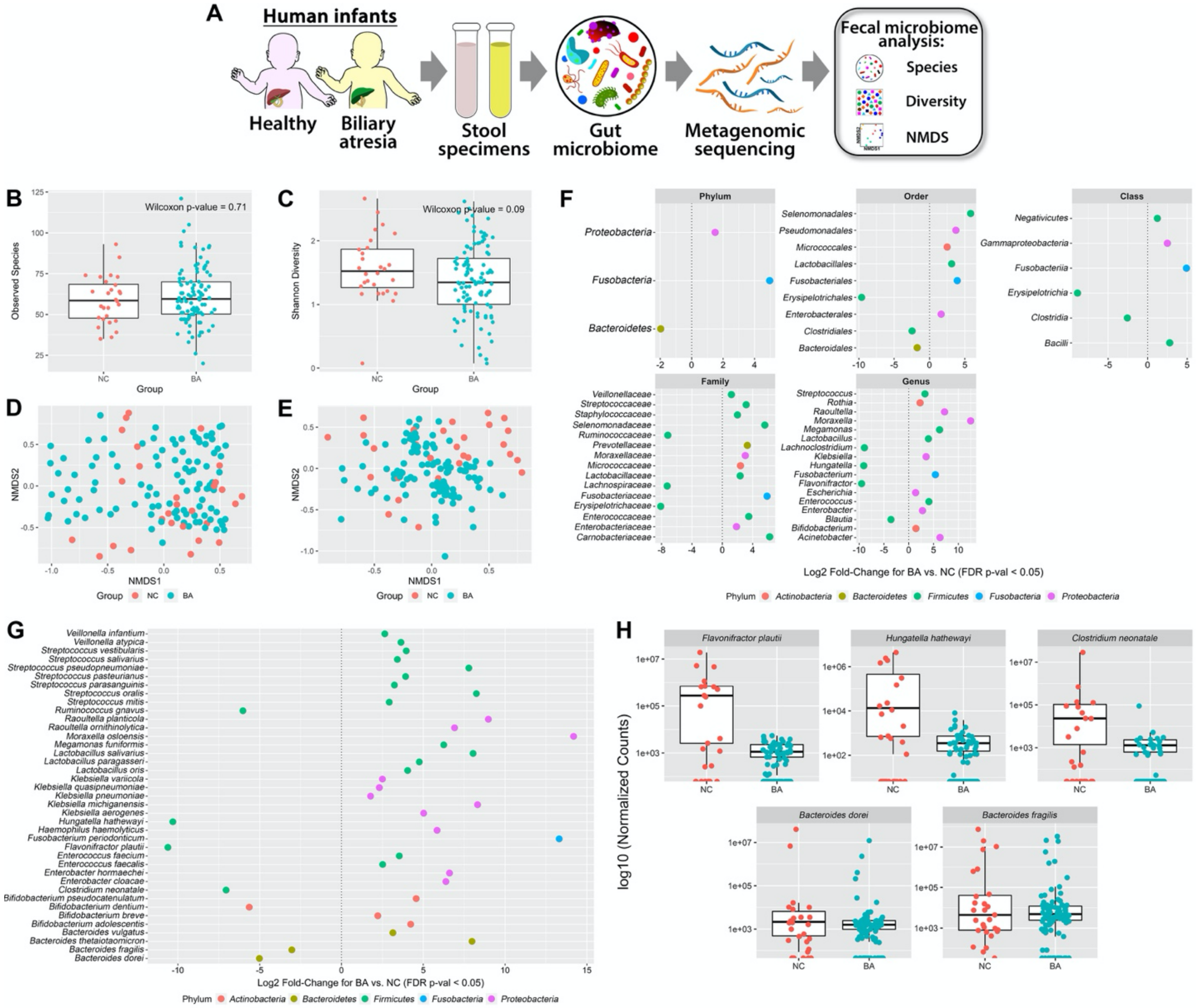

Fig. S7. Gut-microbial diversity and taxonomy of biliary atresia and normal controls.

(**A**) Diagram depicting experimental and analytical strategies. **(B-E**) Number of observed species, Shannon diversity, and non-metric multidimensional scaling ordinations of the Bray-Curtis Dissimilarly and Jaccard Distance from shotgun metagenomic sequencing of stool samples collected from 102 BA infants and 28 normal age-matched controls. The boxes represent the interquartile range (IQR) between the first and third quartiles, and the line

represents the median (2nd quartile). Whiskers within the graph denote the lowest and the highest values within 1.5 x IQR from the first and third quartiles, respectively. Statistical significance was determined using two-sided Wilcoxon rank-sum test. (**F,G**) Log2 fold-changes for differentially abundant taxa estimated from moderated negative binomial regression. (FDR adjusted *p*-value < 0.05). (**H**) Relative abundances of bacterial species with butyrate producing capacity linked to enzymes and genes of the butyrate pathway (shown in Table S6) in stool samples collected from 102 BA infants and 28 normal age-matched controls. The boxes represent the interquartile range (IQR) between the first and third quartiles, and the line represents the median (2nd quartile). Whiskers within the graph denote the lowest and the highest values within 1.5 x IQR from the first and third quartiles, respectively. Statistical significance was determined using two-sided Wilcoxon rank-sum test. Source data for this figure are provided as a Source Data file.

Supplementary Figure 8 (S8)

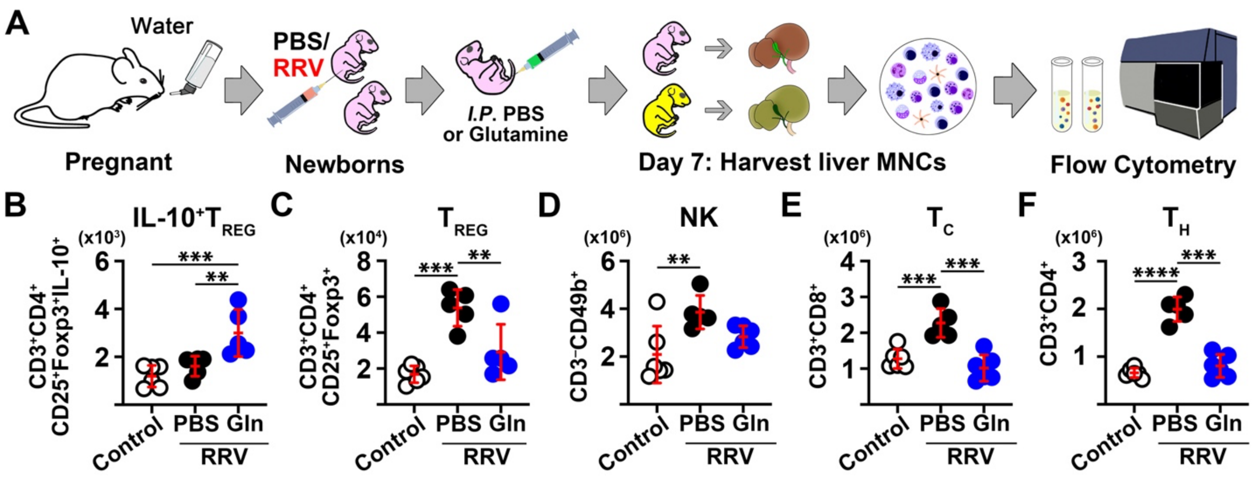

Fig. S8. Glutamine administration increases hepatic IL-10^{+}T$_{REG}$ cells and decreases hepatic effector cells after RRV.

(**A**) Diagram depicting the isolation and flow cytometry evaluation of intrahepatic mononuclear cells from livers of RRV-infected newborn mice treated with PBS or glutamine. (**B**) Flow cytometry analysis of hepatic mononuclear cells from livers of newborn mice injected with RRV or saline show increased IL-10^{+}T$_{REG}$ cells in mice receiving daily intraperitoneal injections of glutamine (Mean ± SD, two-tailed ANOVA with Duncan's multiple comparison, n=5-6 independent neonatal mice per group; $^{**}p<0.01$, $^{***}p<0.001$) and (**C**-**F**) reduced T$_{REG}$, NK, CD8^{+}, and CD4^{+} cells in comparison to PBS-only treatment. Cells harvested 7 days after RRV infection. Mean ± SD, two-tailed ANOVA with Duncan's multiple comparison, n=5-6 per group; $^{**}p<0.01$, $^{***}p<0.001$, $^{****}p<0.0001$). The gating strategy for flow cytometric analysis is shown in Fig. S10A-G. Source data for this figure are provided as a Source Data file.

Supplementary Figure 9 (S9)

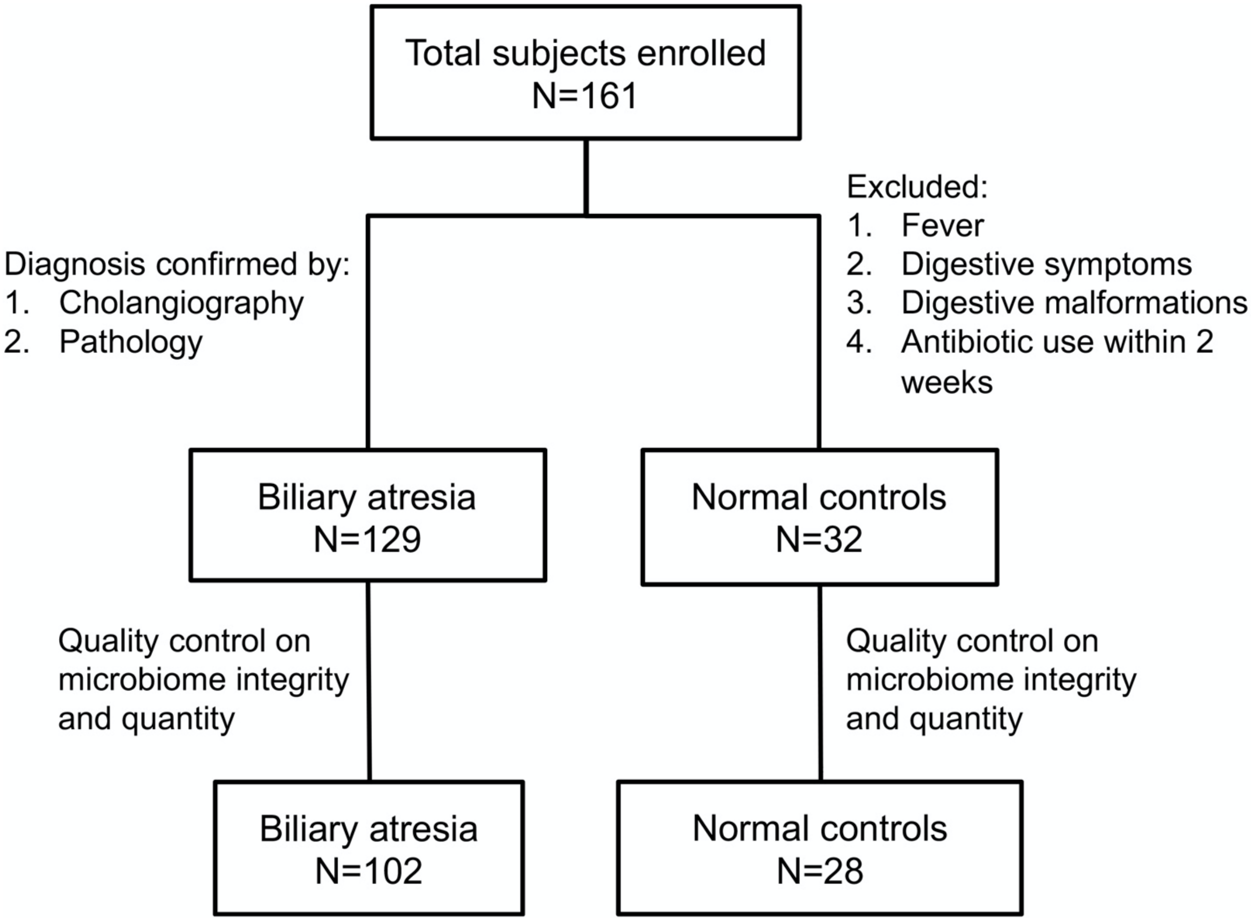

Fig. S9. Flow chart of subject enrollment.

A total of 161 subjects who met the inclusion and exclusion criteria were enrolled, including 129 infants with biliary atresia and 32 controls. After initial quality control for fecal microbiome integrity and quantity, 102 biliary atresia and 28 control specimens satisfied quality criteria and were used for subsequent library construction and metagenomic sequencing.

Supplementary Figure 10 (S10)

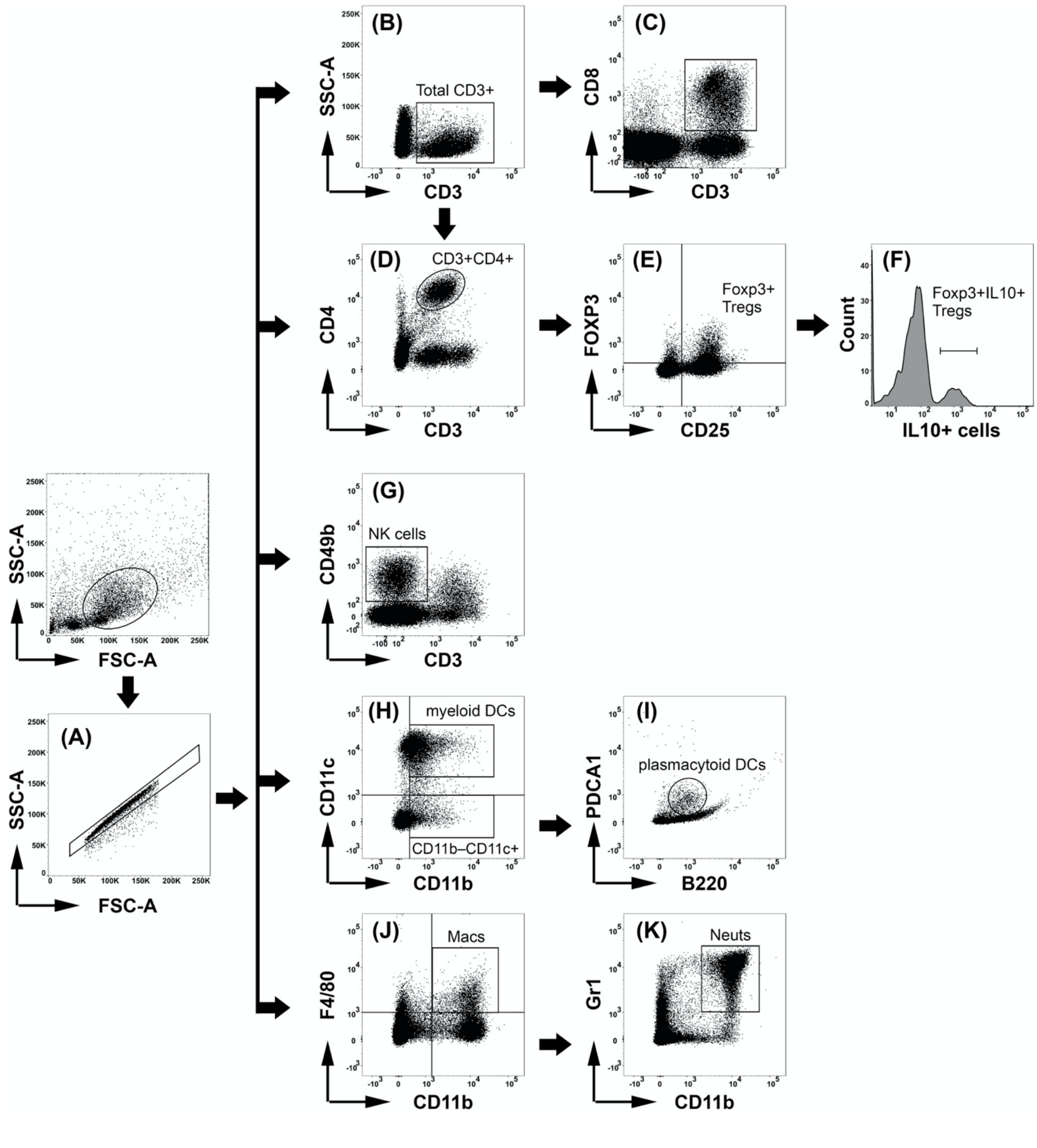

Fig. S10. Representative nested gating strategy for T cells, NK and myeloid cell (dendritic cell, macrophage and neutrophil) populations.

All hepatic immunophenotypes were gated from (A) singlets to (B) CD3+ T cells, (C) cytotoxic T cells (T_C, $CD3^+CD8^+$), (D) helper T cells (T_H, CD3+CD4+), (E) regulatory T cells (T_{REG}, $CD3^+CD4^+CD25^+Foxp3^+$), (F) IL-10-producing T_{REG} ($CD3^+CD4^+CD25^+Foxp3^+IL\text{-}10^+$), (G) NK cells ($CD3^-CD49b^+$), (H) myeloid dendritic cells (mDCs, $CD11b^+CD11c^+$), (I) plasmacytoid dendritic cells (pDCs, $CD11b^-CD11c^+B220^+PDCA1^+$), (J) macrophages ($CD11b^+F4/80^+$), and (K) neutrophils ($CD11b^+Gr1^+$).
