# Supplementary material for: Maternal regulation of biliary disease in neonates via gut microbial metabolites
Source: Nat Commun. 2022 Jan 10;13:18. doi: 10.1038/s41467-021-27689-4 (PMC8748778; doi:10.1038/s41467-021-27689-4)
Supplement: Supplementary file 3 — Reporting Summary [file 41467_2021_27689_MOESM3_ESM.pdf]

nature research

Corresponding author(s): Jorge Bezerra, MD
Shao-tao Tang, MD

Last updated by author(s): Nov 17, 2021

Reporting Summary

Nature Research wishes to improve the reproducibility of the work that we publish. This form provides structure for consistency and transparency in reporting. For further information on Nature Research policies, see our Editorial Policies and the Editorial Policy Checklist.

Statistics

For all statistical analyses, confirm that the following items are present in the figure legend, table legend, main text, or Methods section.

n/a	Confirmed	
☐	☒	The exact sample size (*n*) for each experimental group/condition, given as a discrete number and unit of measurement
☐	☒	A statement on whether measurements were taken from distinct samples or whether the same sample was measured repeatedly
☐	☒	The statistical test(s) used AND whether they are one- or two-sided<br>*Only common tests should be described solely by name; describe more complex techniques in the Methods section.*
☐	☒	A description of all covariates tested
☐	☒	A description of any assumptions or corrections, such as tests of normality and adjustment for multiple comparisons
☐	☒	A full description of the statistical parameters including central tendency (e.g. means) or other basic estimates (e.g. regression coefficient) AND variation (e.g. standard deviation) or associated estimates of uncertainty (e.g. confidence intervals)
☐	☒	For null hypothesis testing, the test statistic (e.g. *F*, *t*, *r*) with confidence intervals, effect sizes, degrees of freedom and *P* value noted<br>*Give P values as exact values whenever suitable.*
☒	☐	For Bayesian analysis, information on the choice of priors and Markov chain Monte Carlo settings
☐	☒	For hierarchical and complex designs, identification of the appropriate level for tests and full reporting of outcomes
☐	☒	Estimates of effect sizes (e.g. Cohen's *d*, Pearson's *r*), indicating how they were calculated

Our web collection on statistics for biologists contains articles on many of the points above.

Software and code

Policy information about availability of computer code

Data collection

The fluorescent signals were detected using a FACSCantoII dual-laser flow cytometer (BD Biosciences).

For NMR metabolomics, one-dimensional 1H-NOESY NMR spectra were acquired on a Bruker Avance II 600 MHz spectrometer using noesygppr1d pulse sequence in the Bruker pulse sequence library. Each spectrum was manually phased, baseline corrected, and referenced to the internal standard TMSP at 0.0 ppm for polar samples using Topspin 3.6 software (Bruker Analytik). Chemical shifts were assigned to metabolites based on the reference spectra found in Human Metabolome Database (HMDB) and Chenomx® NMR Suite profiling software (Chenomx Inc. version 8.4). metabolites were quantified using Chenomx software.

For 16s rRNA sequencing, a combination of best-of-breed analysis packages, including PANDAseq (PAired-eND Assembler for DNA sequences) v2.8 (13), QIIME v1.8 (14), and USEARCH v7.0.1090 (15) were used, and implemented a LONI (USC Laboratory of Neuro Imaging) pipeline workflow for all 16S rRNA pre-processing steps to obtain the OTU tables.

Qiime was used to generate taxonomic assignment and phylogenetic reconstruction from the processed sequence data using python scripts as follow: align_seqs.py script to align the sequences to Greengenes database (v13_8) with NAST alignment algorithm, filter_alignment.py script to remove gap positions in the sequence, make_phylogeny.py script to produce phylogenetic tree from multiple sequence alignment by FastTree, and assign_taxonomy.py script to assign the taxonomy into each sequence with UCLUST algorithm.

For human shotgun metagenomic sequencing, the library was qualified by the Agilent 2100 bioanalyzer and ABI StepOnePlus Realtime PCR System. The qualified libraries were sequenced on Illumina Novaseq 6000 platform (BGI-Shenzhen, China).

No Custom codes were used for analysis.

Data analysis

The flow cytometry data was analyzed using FlowJo software (Tree Star Inc.) version 10.6.
For 16s rRNA sequencing data analysis, abundance of taxa between diseased and resistant neonatal mice was visualized in circular cladograms generated by GraPhlAn. Statistical analyses were performed with STATISTICA 7 (StatSoft, Tulsa, OK), Prism 8 (GraphPad Software, San Diego, CA), SAS 9.3 (SAS Institute, Cary, NC) and PAST 3 software (19).

For human shotgun metagenomic sequencing data analysis, tools from the bioBakery meta'omics analysis environment were used for microbial community taxonomic and functional profiling. Initial quality control was performed using KneadData v0.7.3. Taxonomic profiling with virus detection was performed using MetaPhlAn2 v2.9.5 with the default parameters and v2.9.5 CHOCOPhlAn database to obtain microbial relative abundances for each sample. Functional profiling of gene families and pathways was performed using HUMAnN2 v2.8.1 with the default parameters. The relative abundance of gene families were identified by mapping reads to the UniRef90 protein reference database (https://academic.oup.com/bioinformatics/article/31/6/926/214968) implemented by DESeq2 v1.24.0. Pathway abundances were obtained by aggregation of gene families to MetaCyc pathways. Multi-sample community profiles were generated using the MetaPhlAn2, then integrated into phyloseq v1.28.0 objects for statistical analysis. Differentially abundant MetaCyc pathways were identified using the ANOVA-Like Differential Expression (ALDEX2) package v1.16.0. All metagenomic analyses were performed using the R software environment for statistical computing and graphics v3.6.0.

For manuscripts utilizing custom algorithms or software that are central to the research but not yet described in published literature, software must be made available to editors and reviewers. We strongly encourage code deposition in a community repository (e.g. GitHub). See the Nature Research guidelines for submitting code & software for further information.

Data

Policy information about availability of data

All manuscripts must include a data availability statement. This statement should provide the following information, where applicable:
- Accession codes, unique identifiers, or web links for publicly available datasets
- A list of figures that have associated raw data
- A description of any restrictions on data availability

The 16s rRNA sequencing files and metadata of experimental biliary atresia have been deposited in the European Nucleotide Archive (ENA) at EMBL-EBI and are available under accession number PRJEB40649 (https://www.ebi.ac.uk/ena/browser/view/PRJEB40649).

The raw NMR spectra files of experimental biliary atresia are accessible from MetaboLights (Study Identifier: MTBLS2171, https://www.ebi.ac.uk/metabolights/reviewerabff7e6f-abf1-44cb-976a-9fd380ee712b). Data underlying all figures are provided as Source data files.

The raw metagenomics sequencing reads of human stools and host-phenotype meta-data used in this study are deposited and accessible in the European Genome-phenome Archive data repository with accession code "EGAD00001007735" (https://ega-archive.org/datasets/EGAD00001007735/files). Due to participant confidentiality and informed consent the raw sequencing files are available upon request to data access committee of this collaborative study. The data access committee consists research collaborators from Cincinnati Children's Hospital Center and Tongji Medical College, Huazhong University of Science and Technology. Letter of intent can be submitted to the following primary contacts for restricted access to human sequencing data: Dr. Shao-tao Tang, tshaotao83@hust.edu.cn, and Dr. Jorge Bezerra, Jorge.Bezerra@cchmc.org. The letter of intent shall expect a response from the data access committees within 2-4 weeks. Data access is subject to local rules and regulations. Detailed data use agreement will be shared after letter of intent is received, reviewed, and approved by the data access committee.

All other data related to this article are included in the Supplementary materials and Supplementary tables and are available to the readers. Other information is available from the corresponding authors upon reasonable requests.

Field-specific reporting

Please select the one below that is the best fit for your research. If you are not sure, read the appropriate sections before making your selection.

☒ Life sciences ☐ Behavioural & social sciences ☐ Ecological, evolutionary & environmental sciences

For a reference copy of the document with all sections, see nature.com/documents/nr-reporting-summary-flat.pdf

Life sciences study design

All studies must disclose on these points even when the disclosure is negative.

Sample size

Although no formal power calculations were performed, reasonable sample size estimates were inferred from preliminary experiments. The sample sizes used in our experiments revealed biological differences and ensured sufficient reproducibility of the findings. For all murine experiments, greater than 3 biological replicates were used. In addition to the biological replicates for non-microbiome studies, the experiments were independently repeated at least 2-3 times. In general, the data was routinely collected using independent biological replicates or pooled specimens as needed for the assay/s. We have denoted the number of mice used in each experiment in the Figure legends. For murine microbiome studies, the sample sizes were based on our previous studies (Jee J et al, PLoS One, 2017, 12(8):e0182089).

Data exclusions

The quality filtering in genomic sequencing was done at the time of raw data processing as well as processing for statistical analyses based on the criterion mentioned in the Methods section of the manuscript. Samples that failed to pass these thresholds were excluded from further analyses.

Replication

For the mouse samples, independent experiments were performed at least 3 times to replicate the findings. We collected more than 15 mice

	for phenotypic data, more than 10 mouse for histology, and 3-5 pooled specimens 15-20 mice for serum measurements. For microbiome analyses, we ran duplicate samples across two runs of the microbiome sequencing to maintain reproducibility. All replications were successful.
Randomization	We randomly assigned the mouse for experiment group and control group alternately based on the birthday of the newborns.<br>For human study, we did not do any intervention treatment so there were no randomization required. We prospectively collected information related to major potential covariates of gut microbiome, such as age, sex, and diet, and tested disease groups against controls, using Fisher's exact test. No significant differences were noted (details in Table S6).
Blinding	For the mouse experiments, stool samples were collected, processed, and sequenced in a blinded fashion. Bioinformatic analysis (ESVs and species calling) were also performed in a blinded manner. Investigators were blinded to group allocation during data analysis.<br><br>With regards to the human stool samples, blinding was carried out during the submission of specimens to sequencing protocols as well as at the time of sequencing analysis. To achieve this, all samples were coded in the format of "XX(location code)-XXX (unique numeric ID of subjects)-X (endpoint code)". No group information was revealed at the time of sequencing (handling or processing) or during data analysis. Investigators were blinded to the group allocation/s during data collection. Our location code contains: ET, SZ, XA, XH, ZY, and our unique numeric ID starts from 001 and sequentially randomized to each enrolled subject. Our endpoint code was assigned to letter "A", representing "time at diagnosis".

Reporting for specific materials, systems and methods

We require information from authors about some types of materials, experimental systems and methods used in many studies. Here, indicate whether each material, system or method listed is relevant to your study. If you are not sure if a list item applies to your research, read the appropriate section before selecting a response.

Materials & experimental systems

n/a	Involved in the study
☐	☒ Antibodies
☒	☐ Eukaryotic cell lines
☒	☐ Palaeontology and archaeology
☐	☒ Animals and other organisms
☐	☒ Human research participants
☒	☐ Clinical data
☒	☐ Dual use research of concern

Methods

n/a	Involved in the study
☒	☐ ChIP-seq
☐	☒ Flow cytometry
☒	☐ MRI-based neuroimaging

Antibodies

Antibodies used	FITC-conjugated anti-mouse CD3 (clone: 17A2, eBioscience, Catalog # 11-0032-82). Dilution: 1/200.<br>Pacific Blue-conjugated anti-mouse CD4 (clone: RM4-5, BioLegend, Catalog # 100531). Dilution: 1/100.<br>APC-conjugated anti-mouse CD8a (clone: 53-6.7, eBioscience, Catalog # 17-0081-81). Dilution: 1/400.<br>PE/Cy7-conjugated anti-mouse CD25 (clone: 7D4, SouthernBiotech, Catalog # 1595-17). Dilution: 1/50.<br>PerCp/Cy5.5-conjugated anti-mouse CD3 (clone:17A2, BioLegend, Catalog # 100218). Dilution: 1/20.<br>FITC-conjugated anti-mouse/rat Foxp3 (clone: FJK-16s, eBioscience, Catalog # 11-5773-82). Dilution: 1/50.<br>PE-conjugated anti-mouse IL-10 (clone: JES5-16E3, eBioscience, Catalog # 12-7101-41). Dilution: 1/20; 5μl/test.<br>APC-conjugated anti-mouse CD49b (clone: DX5, BioLegend, Catalog # 108910). Dilution: 1/80.<br>PerCp/Cy5.5-conjugated anti-mouse CD11b (clone: M1/70, BioLegend, Catalog # 101228). Dilution: 1/80.<br>APC/Cy7-conjugated anti-mouse Gr-1 (clone: RB6-8C5, BioLegend, Catalog # 108424). Dilution: 1/80.<br>Pacific Blue-conjugated anti-mouse F4/80 (clone: BM8, BioLegend, Catalog # 123124). Dilution: 1/200.<br>FITC-conjugated anti-mouse CD11c (clone: N418, BioLegend, Catalog # 117306). Dilution: 1/200.<br>APC-eFluor® 780-conjugated anti-human/mouse B220 (clone: RA3-6B2, eBioscience, Catalog # 47-0452-82). Dilution: 1/40.<br>APC-conjugated anti-mouse PDCA-1 (clone: JF05-1C2.4.1, Miltenyi Biotec, Catalog # 130-123-789). Dilution: 1:50; 2μl/test.<br><br>Note: Dilutions for all the antibodies are calculated for up to 1x10^6 cells/100μl of the staining volume.<br><br>Intracellular staining for Foxp3 and IL-10 were performed using a Foxp3/Transcription Factor Staining Buffer Set (Life Technologies Corporation, 00-5523-00).
Validation	All antibodies used in this study are from commercial sources and have been validated by the respective vendors. The validation data for each antibody is available on the corresponding manufacturer's website and provides extensive documentation of published articles citing the use of their products, relevant dilutions, applications, etc. Catalog numbers and name of the manufacturer for each antibody is provided in the "Antibodies used" section of this Reporting Summary.

Animals and other organisms

Policy information about studies involving animals; ARRIVE guidelines recommended for reporting animal research

Laboratory animals	All animal studies were performed in strict accordance with the recommendations of the Institutional Animal Care and Use

	Committee of Cincinnati Children's Hospital Medical Center (Approved IACUC protocol: IACUC2020-0006; PI: Pranavkumar Shivakumar). Breeding pairs of adult BALB/c mice were obtained from Charles River Laboratories, acclimated and maintained in microisolator cages in a specific pathogen-free facility. The mice were housed in a room equipped with a 12-hour dark light cycle and temperature and humidity maintained between 65-75ºF and 40-60%, respectively. Mice had free access to water and sterilized chow (Purina LabChows Rodent Laboratory Chow 5010; Protein: 23.0%, Fat: 4.5%, Fiber: 6.0%) and were monitored daily by qualified veterinary staff to assure humane conditions. Biliary atresia was induced in neonatal BALB/c mice within 24 hours of birth by intraperitoneal injection with 1.5 x 106 ffu of RRV in a 20 µl volume; a similar volume of 0.9% saline solution was used as controls. Infected mice were monitored daily for jaundice, acholic stool, weight and mortality until 2 weeks of life. Sex determination of newborn mice was not performed due to equal susceptibility of male and female pups. Although the anogenital distance has been described to sex neonatal mice, about 50% of newborn mice can be wrongly classified and therefore unreliable.
Wild animals	The study does not include wild animals.
Field-collected samples	The study does not include samples collected from the field.
Ethics oversight	All studies were performed in strict accordance with the recommendations of the Institutional Animal Care and Use Committee of Cincinnati Children's Hospital Medical Center (Approved IACUC protocol: IACUC2020-0006).

Note that full information on the approval of the study protocol must also be provided in the manuscript.

Human research participants

Policy information about studies involving human research participants

Population characteristics	Full population characteristics are provided in the manuscript in Online material and methods, and in Supplementary Fig. S9 and Table S6.
Recruitment	Subjects with biliary atresia were enrolled in a non-interventional prospective of study from November 2017 until July 2019; age-matched participants without liver diseases were also enrolled to serve as controls. Stools were collected at the time of clinical diagnosis prior to surgery. The demographic characteristics including age, sex, diet, and liver biochemical parameters are included in Suppl. Table S6. The inclusion criteria required confirmative diagnosis of biliary atresia by intraoperative cholangiography and demonstration of fibrosing obstruction of extrahepatic bile ducts. The selection criteria for age-matched controls included age between 2 weeks and 6 months, no fever, no liver disease, no digestive symptoms such as constipation and diarrhea at the time of stool collection, and no antibiotic administration within 2 weeks.
Ethics oversight	Consent and agreement were obtained from parents or legal guardians to participate in our research. The multicenter study followed the ethical guidelines of the 1975 Declaration of Helsinki as reflected in the approval by the institutional review board and ethics committee at each participating center. Study protocols were approved by the institutional review board (IRB) and ethics review committee of the following institutions/centers: Department of Pediatric Surgery, Union Hospital, Tongji Medical College, Huazhong University of Science and Technology, Wuhan, Hubei, 430022, China; Department of Neonatal Surgery, Xi'an Children's Hospital, Xi'an, Shanxi, 710003, China; Department of Pediatric Surgery, Wuhan Children's Hospital, Tongji Medical College, Huazhong University of Science and Technology, Wuhan, Hubei, 430015, China; Department of General Surgery, Shenzhen Children's Hospital, Shenzhen, Guangdong, 518038, China; Department of Pediatric General Thoracic and Urology Surgery, The Affiliated Hospital of Zunyi Medical University, Zunyi, Guizhou, 563000, China; Department of Pediatric Surgery, Jiangmen Maternity and Child Health Care Hospital, Jiangmen, Guangdong, 529000, China.

Note that full information on the approval of the study protocol must also be provided in the manuscript.

Flow Cytometry

Plots

Confirm that:

- [x] The axis labels state the marker and fluorochrome used (e.g. CD4-FITC).
- [x] The axis scales are clearly visible. Include numbers along axes only for bottom left plot of group (a 'group' is an analysis of identical markers).
- [x] All plots are contour plots with outliers or pseudocolor plots.
- [x] A numerical value for number of cells or percentage (with statistics) is provided.

Methodology

Sample preparation	Single cell suspension were made from hepatic mononuclear cells (MNCs) obtained from livers of neonatal mice 7 days after challenge with saline or RRV using density gradients of Percoll.
Instrument	BD FACSAria II and BD Influx Systems (2350 Qume Drive, San Jose, CA 95131-1807, USA)
Software	FlowJo v10.6. (BD Biosciences)
Cell population abundance	1-2 million antigen-specific CD4+ T cells were sorted per group.
Gating strategy	The cells were first gated on the lymphocyte population on the FSC/SSC plot. Singlets were gated on FSC-A/FSC-W plot. Thereafter myeloid and lymphoid cells were determined based on staining for surface marker or intracellular marker.

Subsequent characteristics were analyzed within these population.

☒ Tick this box to confirm that a figure exemplifying the gating strategy is provided in the Supplementary Information.
